# Characteristics of a Magnetic Field Sensor with a Concentrating-Conducting Magnetic Flux Structure

**DOI:** 10.3390/s19204498

**Published:** 2019-10-17

**Authors:** Xuelei Li, Xiaofeng Zhao, Dianzhong Wen

**Affiliations:** School of Electronics Engineering, Heilongjiang University, Harbin 150080, China; 20141043@s.hlju.edu.cn (X.L.); wendianzhong@hlju.edu.cn (D.W.)

**Keywords:** concentrating-conducting magnetic flux structure, Hall element, finite element simulation

## Abstract

A magnetic field sensor with a new concentrating-conducting magnetic flux structure (CCMFS) is proposed in this paper, using a silicon-on insulator (SOI) Hall element fabricated by complementary metal oxide semiconductor (CMOS) technology as a magnetic sensitive unit. By fixing the CCMFS above the Hall element packaged on a printed circuit board (PCB) based on inner-connect wire bonding technology, a non-magnetized package can subsequently be obtained. To analyze the inner magnetic field vector distribution of the CCMFS, a simulation model was built based on a finite element software, where the CCMFS was processed using Ni-Fe alloys material by a low speed wire-cut electric discharge technology. The test results showed that the measurement of magnetic fields along a sensitive and a non-sensitive axis can be achieved when *V*_DD_ = 5.0 V at room temperature, with magnetic sensitivities of 122 mV/T and 132 mV/T in a testing range from −30 mT to 30 mT, respectively. This study makes it possible to not only realize the detection of magnetic field, but also to significantly improve the sensitivity of the sensor along a non-sensitive axis.

## 1. Introduction

In recent years, increasing attention has been paid to improving the performance of magnetic field sensors on account of the development of micro-electromechanical systems (MEMS) technology and complementary metal oxide semiconductor (CMOS) technology. It is well known that a magnetic field sensor has a certain magnetic sensitive direction according to the principle of that sensor. For instance, magnetic sensitive transistors (MST) are sensitive to the external magnetic field parallel to the sensor chip surface, Hall elements measure the external magnetic field perpendicular to the magnetic sensitive layer [1,2]. In addition, the measurement of multi-directional magnetic field vectors is in high demand in different application fields such as navigation, the automotive industry and deep-sea exploration [3]. To meet the requirements of these applications, space magnetic field vector sensors are assembled and formed by multi same single-axis magnetic field sensors, with a good uniformity of magnetic sensitivity. However, the development of magnetic field sensors has been limited due to sensitivity cross-interference. So, two methods have been proposed to overcome the above limitation and achieve measurement along the non-sensitive axis, including structural optimization and external magnetic field modulation. In terms of the external magnetic field modulation, researchers have focused on adjusting the direction of external magnetic flux by especially tailored magnetic material structure where the magnetic flux guides (or conducting magnetic flux structure) are designed for magnetic field sensors to detect the magnetic field vector along the non-sensitive axis [4,5,6,7,8]. Meanwhile, magnetic concentrators (or concentrating magnetic flux structure) have been studied to improve the conversion efficiency of magnetic flux, obtain high magnetic sensitivity and detect weak magnetic field. There are two main factors affecting magnetic field gain, i.e., structural shape and soft magnetic material permeability. Magnetic concentrators with different shapes such as a T-shape, bar shape and triangle shape, etc., but which are processed by the same materials have different collecting efficiency in the detection of external magnetic fields [9,10,11,12,13,14]. Moreover, typical soft magnetic materials such as Ni, Co and their alloys, have been adopted to fabricate concentrators because of their excellent saturation magnetic flux density (*B*_s_), high relative permeability (*μ*_r_), low coercivity (*H*_c_) and remanence magnetic flux density (*B*_r_). However, it is hard to realize a highly sensitive measurement by using the proposed structure of magnetic field sensors along a non-sensitive axis at present due to their poor direction adjustment of the magnetic field and loss compensation of the magnetic flux during the conduction process.

In this study, to overcome the above limits and to realize a high magnetic sensitivity measurement along a non-magnetic sensitive direction, a magnetic field sensor with a new type of concentrating-conducting magnetic flux structure (CCMFS) consisting of a Hall element and a CCMFS was designed and fabricated. On the basis of the theoretical analysis of CCMFS, the conduction mechanism of the proposed sensor is discussed in detail and its properties of magnetic flux concentration and conduction of CCMFS were simulated. The magnetic field sensor with CCMFS was packaged on a printed circuit board (PCB). The magnetic sensitive characteristics of this magnetic field sensor are tested and analyzed at room temperature. This study provides a novel approach to detect external magnetic field along the non-magnetic sensitive direction and improve the magnetic sensitivity, which could have a significant impact on the application of magnetic sensors in future.

## 2. Structure and Principle

### 2.1. Basic Structure

**Figure 1:**
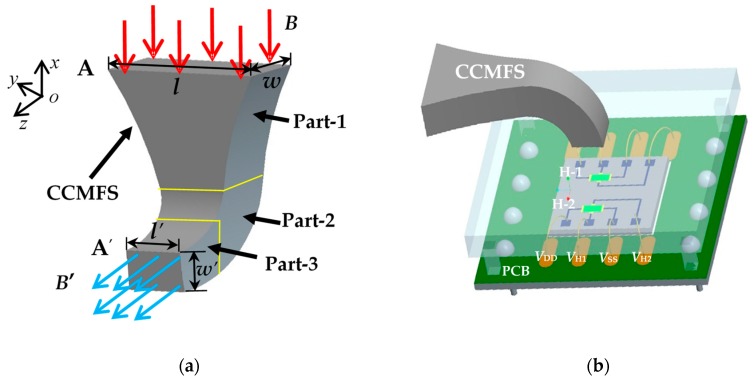
The basic structure of the magnetic field sensor: (**a**) CCMFS; (**b**) magnetic field sensor with Hall element and CCMFS.

Figure 1a shows the basic structure of the proposed concentrating-conducting magnetic flux structure (CCMFS). The CCMFS with an L-shaped structure is divided into three parts, i.e., part-1, part-2 and part-3, which are characterized by their gradually decreasing cross-section area The magnetic flux import and export surfaces are called surface-A and surface-A’, the external magnetic flux was collected at surface-A and then redirected at surface-A’. As seen in Figure 1a, the lengths of surface-A and surface-A’ are named as *l* and *l*’, and the widths of surface-A and surface-A’ are taken as *w* and *w*’ respectively. The basic structure of the proposed magnetic field sensor is shown in Figure 1b, consisting of a silicon-on insulator (SOI) Hall element, a CCMFS and a PCB with metal electrodes. The SOI Hall element with a magnetic sensitive layer, two control current electrodes (*V*_DD_, *V*_SS_) and two Hall output probes (*V*_H1_, *V*_H2_) was used as the magnetic sensitive unit. Using CMOS technology fabricates two sensing elements (H-1, H-2) symmetrically on the same chip. By pasting the chip on the PCB, the CCMFS was fixed above the magnetic sensitive layer of the Hall element with surface-A perpendicular to the chip surface and surface-A’ parallel to the chip surface. 

### 2.2. Theoretical Analysis of CCMFS 

As shown in Figure 1a, when the CCMFS is placed in a uniform magnetic field along the *x*-axis direction perpendicular to surface-A, the magnetic flux will be concentrated and expanded toward the peak at part-1, change conduction direction at part-2 and decline at part-3.Then, the magnetic flux along *x*-axis will appear at surface-A’ as the magnetic flux along the *z*-axis, where the magnetic flux density (*B*) along the non-sensitive axis is converted into the magnetic flux density (*B*’) along the sensitive axis of the Hall element by the CCMFS. To achieve a balance between magnetic field amplification and guiding efficiency, the design of the CCMFS was optimized, where the reasonable working range of CCMFS is defined as the value of the external magnetic flux density from 0 T to the saturation magnetic flux density of soft magnetic materials. 

In a vacuum environment, based on the definition of magnetic flux density [11], the equation of *B* can be given as:

(1) $$B=\mu_{0}H_{0}$$

where *H*_0_ is the external uniform magnetic field intensity and vacuum magnetic permeability *μ*_0_ = 4π × 10^−7^ N/A^2^.

When the high-permeability CCMFS is placed into an external uniform magnetic field, the magnetic flux can be attracted and collected, resulting in concentrating the external magnetic flux through CCMFS at surface-A and representing a converting value of magnetic flux density *B*’ at surface-A’. According to international units [11], the sum of *B*’, *H*’ and magnetization *M* (the vector dipole moments per unit volume) conforms to the following relationship as:

(2) $$B^{\prime }=\mu_{0}\cdot \left(H^{\prime }+M\right)$$

Based on the definition of magnetization [15], the relationship of *M* and the volume magnetic susceptibility *x_v_* is given as:

(3) $$M=x_{v}H^{\prime }$$

where $x_{v}=\mu_{r}-1$, and *μ*_r_ is the relative permeability. 

According to the definition of relative permeability [11], *μ*_r_ can be given as the following:

(4) μr=μμ0

where *μ* is the permeability, i.e., the ratio of *B* and *H* at any point on a material hysteresis loop. 

On the basis of Equations (2)–(4), *B*’ at the surface-A’ can be expressed as:

(5) $$B^{\prime }=\mu_{0}\cdot \left(H^{\prime }+M\right)=\mu_{0}\cdot \left(H^{\prime }+x_{v}H^{\prime }\right)=\mu_{0}\cdot \left(1+x_{v}\right)\cdot H^{\prime }$$

When the CCMFS is magnetized, the internal CCMFS would generate a demagnetizing component *H*_d_ inverse to *H*_0_ [11,16]. *H*’ is the sum of *H*_0_ and *H*_d_, and is shown as follows:

(6) $$H^{\prime }=H_{0}+H_{d}$$

For this CCMFS, the relationship between *H*_d_ and *M* can be expressed as:

(7) $$H_{d}=-N_{k}\cdot M$$

where N_k_ is the demagnetizing factor [9,16]. By calculating, *H*’ can be given as:

(8) $$H^{\prime }=\frac{H_{0}}{1+N_{k}x_{v}}$$

The N_k_ is defined by:

(9) $$N_{k}=\frac{w^{\prime }l^{\prime }}{L^{2}}\left(\ln\frac{4L}{w^{\prime }+l^{\prime }}-1\right),(N_{k}>0)$$

where *L* is the length of the conduction path [9,11,17]. The inner magnetic field of CCMFS is affected by N_k_, depending on the object structure. Besides, we defined *A*_m_ as the magnetic field conduction and amplification factor. In accordance to the above analysis, the relative equation between *B* and *B*’ can be given:

(10) $$B^{\prime }=A_{m}\cdot B$$

From Equations (2)–(10), the *A*_m_ is expressed as:

(11) $$A_{m}=\frac{B^{\prime }}{B}=\frac{\mu_{0}\cdot \left(1+x_{v}\right)\cdot H^{\prime }}{\mu_{0}H_{0}}=\frac{\mu_{r}}{1+N_{k}\left(\mu_{r}-1\right)}$$

From Equation (11), it can be seen that *A*_m_ is relative to N_k_ and *μ*_r_, thus it is necessary to improve the performance of the CCMFS by optimizing the structure. Based on the above analysis, it is possible to adjust the magnetic flux direction properly and conduct the magnetic flux effectively using the CCMFS during the modulation process. This is important for studying the principle of the proposed magnetic field sensor.

### 2.3. Operating Principle of Magnetic Field Sensor

**Figure 2:**
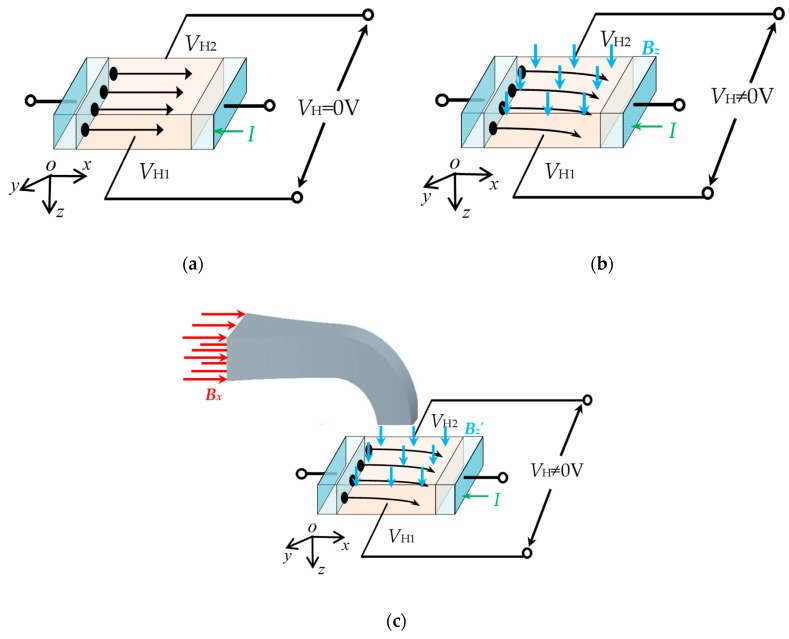
The working principle of the magnetic field sensor (the Hall element and CCMFS are appropriately enlarged at different zoom factors): (**a**) *B* = 0 T; (**b**) *B_z_* ≠ 0 T; (**c**) *B_x_* ≠ 0 T.

To observe the electronic transportation clearly and analyze the working principle of the magnetic field sensor, Figure 2 gives the enlarged view of the Hall element and the CCMFS at different zoom factors. Under ideal conditions, an equal value for *V*_H1_ and *V*_H2_ can be obtained because the electronics transportation of the Hall element is not affected by Lorentz force under no external magnetic field, as shown in Figure 2a. Figure 2b shows the electronics deflection activated by Lorentz force when applying an external magnetic field *B* along the sensitive axis (*z*-axis) of the Hall element. At this time, the output voltage *V*_H_ is defined as the difference value between *V*_H1_ and *V*_H2_. On the basis of the Hall effect [18], the expression of *V*_H_ and *B_z_* can be given under a constant voltage source:

(12) VH=f′H(LH,θ)·μn·VDD·Bz

where *θ* is the Hall angle, *μ*_n_ is the electronic mobility in a magnetic sensitive layer, *V*_DD_ is the supply voltage, f′H(LH,θ) is the geometric structure factor.

As can be seen in Figure 2c, the Hall element sensing to the magnetic field component along the non-sensitive axis (*x*-axis) results in a difference of potential between the two Hall output probes due to the effect of CCMFS on *B_x_*. According to Equation (10) and the coordinate system in Figure 2c, the relative equation of *B_z_*’ and *B_x_* can be expressed:

(13) $$B_{z}^{\prime }=B_{x}\cdot A_{m}$$

By substituting Equation (13) into Equation (12), the expression between *V*_H_ and B*_x_* is shown as:

(14) VH=f′H(LH,θ)·μn·VDD·Bz′=f′H(LH,θ)·μn·VDD·Bx·Am

Based on the definition of sensitivity [17], the magnetic sensitivity *S_x_* along the non-sensitive axis (*x*-axis) of the magnetic field sensor is defined as the Hall output voltage *V*_H_ per unit magnetic field *B_x_* and written as following:

(15) Sx=VHBx=f′H(LH,θ)·μn·VDD·Am

In terms of the analysis, it is possible to achieve the detection of the magnetic field along non-sensitive axis by using CCMFS. From Equation (15), *S_x_* is proportional to *A*_m_. Theoretical analysis results show that the optimally designed CCMFS can clearly enhance the performance of a magnetic field sensor.

## 3. Simulation Analysis and Fabrication Technology

### 3.1. Characteristics Simulation of CCMFS 

**Figure 3:**
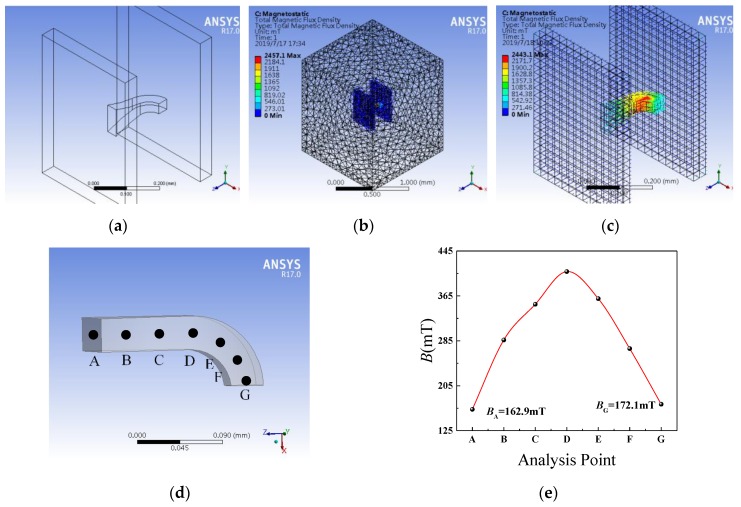
The simulation of inner magnetic flux distribution of CCMFS by using finite element software: (**a**) CCMFS simulation model; (**b**) the simulated enclosing space magnetic field environment; (**c**) inner magnetic flux distribution of CCMFS; (**d**) typical analysis points selected on the center of cross sections along analysis paths; (**e**) relationship curves between magnetic flux of analysis points and the points.

Assuming that the soft magnetic material of CCMFS has a linear *B*–*H* relation curve, the inner magnetic flux distribution of CCMFS in a uniform magnetic field is solved by the Workbench module of ANSYS 17.0. In the following simulations, the simulated coercivity and remanence of the uniform magnetic field model are 8 × 10^5^ A/m and 0.5 T, respectively. The simulated relative permeability of Ni-Fe alloys CCMFS is 3000. Figure 3a shows the simulation model consisting of the uniform magnetic field model and CCMFS. The uniform magnetic field model is composed of two cuboid plates, and is able to provide the magnetic field for about 0.15 T. The CCMFS is placed in the center of the two plates and the inner magnetic flux distribution of CCMFS in this uniform magnetic field is studied. The simulated size of surface-A and surface-A’ are 100 × 35 μm^2^ and 35 × 35 μm^2^, respectively. The enclosing space magnetic field environment and the result of the magnetic flux distribution inner CCMFS are shown in Figure 3b,c. Through analysis paths we calculate the value of magnetic flux from surface-A to surface-A’ of the CCMFS. We defined seven points from A to G, which are the central points of the cross section along the analysis path. After fitting and calculating, the relation curves between the magnetic flux of analysis points and the points are shown in Figure 3e. The inner magnetic flux distribution of CCMFS has a tendency to climb up (at part-1), change direction (at part-2) and then decline (at part-3). The value of the magnetic flux at surface-A’ has improved approximately 10% compared to the surface-A. Finite element analysis simulation results prove that CCMFS can adjust the direction of the magnetic field vector. 

**Figure 4:**
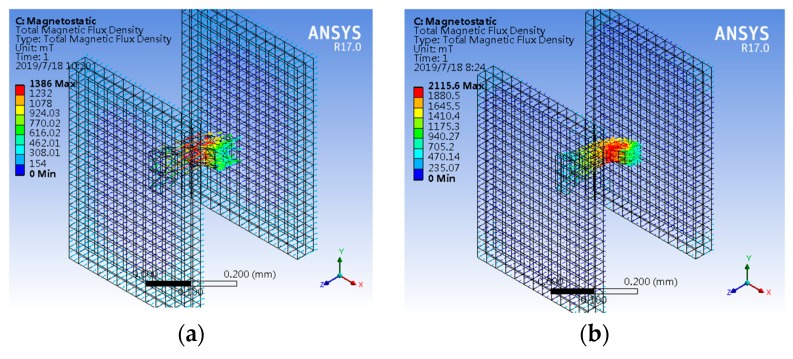

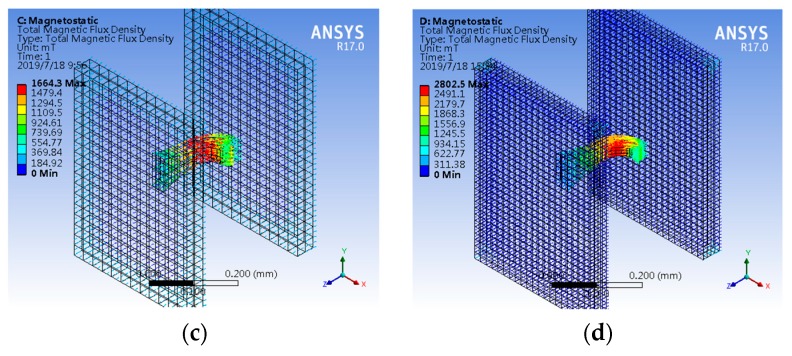
The inner magnetic flux distribution simulation for CCMFS with different export surface sizes: (**a**) 50 × 50 μm^2^; (**b**) 20 × 20 μm^2^; (**c**) 40 × 40 μm^2^; (**d**) 30 × 30 μm^2^.

To study the effect of the export surface (surface-A’) size on the modulating performance of the magnetic field, different sizes of surface-A’ such as 50 × 50 μm^2^, 20 × 20 μm^2^, 40 × 40 μm^2^ and 30 × 30 μm^2^ were designed and investigated, as shown in the simulation results in Figure 4a–d. It can be seen that in the condition of max magnetic flux density, excellent magnetic field concentration and conduction appear at surface-A’ with the size of 30 × 30 μm^2^. The magnetic concentration performance decreased due to the oversize area of surface-A’. In addition, the magnetic flux loss in the process of conduction is increased, and the magnetic modulation performance is reduced due to surface-A’ being too small. The simulation analysis indicates it is possible to optimize the design of CCMFS with different surface-A’ sizes.

**Figure 5:**
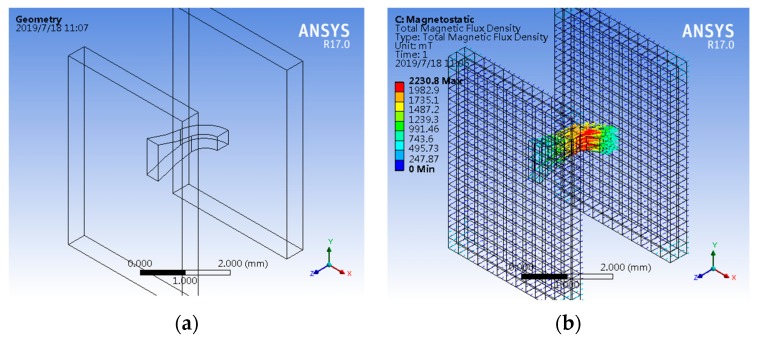
The simulation of inner magnetic flux distribution with the size of the CCMFS model enlarged 10 times compared to Figure 3a: (**a**) CCMFS simulation model; (**b**) magnetic flux distribution in the enlarged CCMFS model.

To satisfy the actual manufacturing and packaging process, a set of larger, comparative simulations corresponding to Figure 3a is carried out as shown in Figure 5a,b. The size of surface-A and surface-A’ are 1000 × 350 μm^2^ and 350 × 350 μm^2^, respectively. The simulation model is about 10 times bigger than the original model. Simulation results show that when the CCMFS is enlarged on an equal scale, the modulation performance of magnetic flux is not significantly affected. 

**Figure 6:**
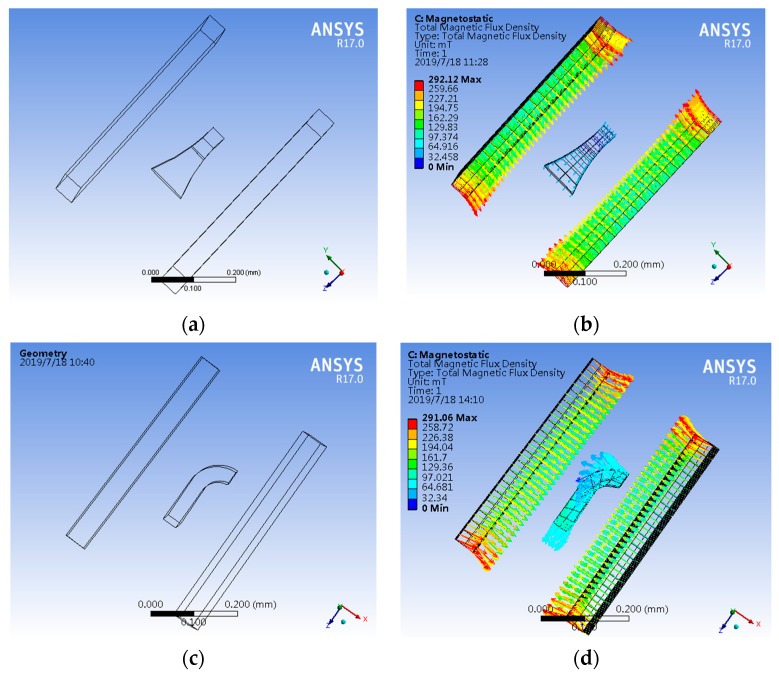
The inner magnetic flux distribution simulation for CCMFS under the external magnetic field vector: (**a**) CCMFS simulation model-1 under the magnetic field vector orthogonal to the normal line of export surface; (**b**) magnetic flux distribution inner CCMFS model-1; (**c**) CCMFS simulation model-2 under the magnetic field vector parallel to the normal line of export surface; (**d**) magnetic flux distribution in CCMFS model-2.

To study the effect of the uniform magnetic field along different vector directions on the performance of the magnetic flux conduction in the comparative simulation analysis, the magnetic field vector was applied to the side surface of CCMFS along different axes. Two sets of simulation models and results are shown in Figure 6a–d. Figure 6a,c show the comparison between simulation model-1 and model-2, which consist of the uniform magnetic field model and CCMFS. Figure 6b,d show the comparison between simulation results of the magnetic flux distribution in the two models. When the magnetic field is applied perpendicularly to the normal line of surface-A’ and parallel to the normal line of surface-A’ of CCMFS, no obvious collection and conduction of the magnetic flux in CCMFS was observed. This proves that the CCMFS can perform normally only when importing the magnetic flux from surface-A, except for that entering from the other surfaces. By comparing the simulation results, the directional characteristic of magnetic flux conduction is proven to exist.

### 3.2. Fabrication Technology 

**Figure 7:**
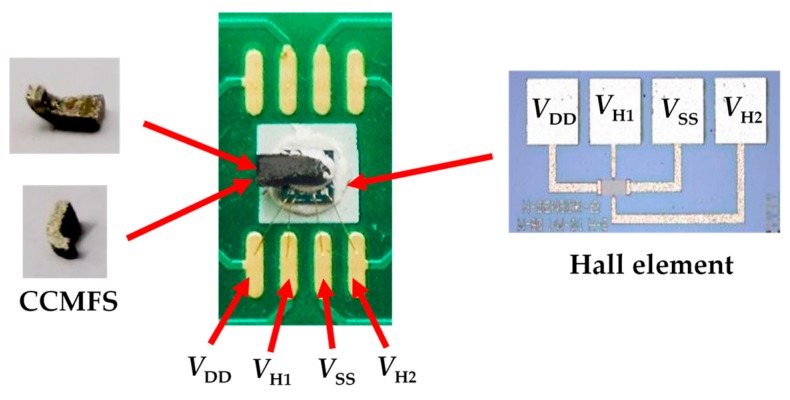
The photo of the proposed sensor (insets are the CCMFS and Hall element, respectively).

To study the modulation performance of CCMFS for the external magnetic field along the non-sensitive axis of the Hall element, the chip of the Hall element was designed and fabricated on a SOI wafer (device layer with n-type, ρ = 1 Ω/cm) with <100> orientation by using CMOS technology, where the CCMFS was machined using Ni-Fe alloys material on the basis of low speed wire-cut electric discharge technology. The main fabrication technology processes were as follows: (a) a SiO_2_ layer with a thickness of 550 nm was grown on a cleaned surface of SOI wafer by adopting thermal oxidation; (b) first photolithography was used to form a n^-^ region as a magnetic sensitive layer, and then a thin oxygen film of 50 nm was grown as a buffer layer and P-ion was injected with a dose and energy of 2E13 cm^−2^ and 60 keV; (c) second photolithography was used to form a n^+^ region used as control current electrodes and Hall voltage output poles, respectively. Thereafter, P-ion was injected with an injection dose and energy of 3E15 cm^−2^ and 60 keV, and the impurity atoms were activated using a subsequent annealing treatment; (d) third photolithography was performed to form contact holes, followed by sputtering Al film on the top surface by using magnetron-sputtering technology, and then the Al layer was etched to develop metal interconnect lines and electrodes, finally the chip was metalized at 420 °C for 25 min to achieve an ohmic contact; (e) low speed wire-cut electric discharge technology was used to form an approximate shape; (f) the specific shape was burnished to secure an accurate proportion of surface-A and surface-A’; (g) the surfaces were treated and polished to eliminate the severe oxidation and obtain a fine scratch; (h) the surface of CCMFS was cleaned and inner-connect wire bonding technology was used to achieve a non-magnetized package of the Hall element by fixing the CCMFS on a PCB. The photos of the insets of the CCMFS and Hall element are shown in Figure 7, where the area of the Hall element chip is 1.5 × 1.5 mm^2^, the area of the Hall magnetic sensitive layer is 80 × 40 μm^2^, and the size of surface-A and surface-A’ for the CCMFS are 1000 × 350 μm^2^ and 350 × 350 μm^2^, respectively.

## 4. Results and Discussion

To analyze the effect of the CCMFS on the characteristics of the proposed magnetic field sensor, a testing system were constructed, consisting of a high precision magnetic field generating system (CH-100), a digital multimeter (Aglient 34410A), a programmable linear direct-current (DC) power source (RIGOL DP832A), and a high-low temperature experiment chamber (GDJS-100G). The measurement of the temperature characteristics of the proposed sensor can be carried out in the range from −40 °C to 85 °C.

### 4.1. Magnetic Sensitive Characteristic of Hall Elements

**Figure 8:**
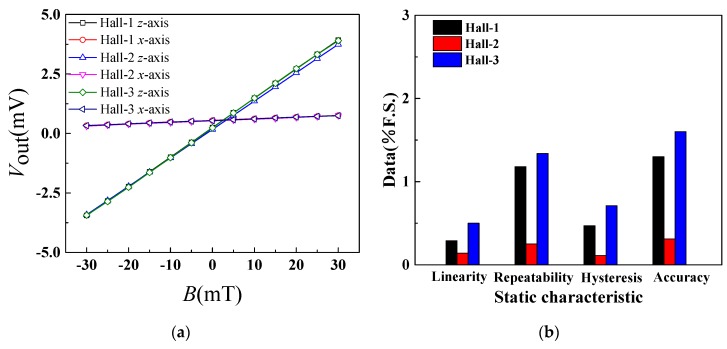
The magnetic sensitive characteristics of three Hall elements: (**a**) the relationship curves between *V*_out_ and *B*; (**b**) the static characteristic histograms of Hall-1, Hall-2 and Hall-3.

To study the magnetic sensitive characteristics of the improved sensor, the performance of the Hall elements before packaging the CCMFS was investigated by repeated testing (three times) to the positive and negative strokes. The test result indicates that when *V*_DD_ = 5.0 V at room temperature, the average value of three Hall elements *V*_out_ can be obtained under an external magnetic field along the sensitive axis (*z*-axis) and the non-sensitive axis (*x*-axis) from −30 mT to 30 mT with a step of 5 mT. The relationship curves between *V*_out_ and *B* along the *z*-axis and *x*-axis are shown in Figure 8a, and the static characteristic histograms including linearity, repeatability, hysteresis and accuracy of Hall-1, -2 and -3 along the *z*-axis are shown in Figure 8b. At a constant operating voltage, the absolute value of *V*_out_ increased with the increasing *B* in the positive and the negative magnetic field directions along a sensitive-axis. The sensitivities of Hall-1, -2 and -3 are 122.8 mV/T, 119.4 mV/T and 122.3 mV/T along the *z*-axis, and are 7.1 mV/T, 7.3 mV/T and 6.9 mV/T along the *x*-axis, respectively. According to the static characteristic histogram, the above-mentioned characteristics of Hall-2 are less than the others, meaning that the Hall-2 has a better static property compared to the other samples, with an accuracy of 0.31% F.S. 

### 4.2. Characteristic Detection of the Magnetic Field Sensor along the Non-Sensitive Axis

**Figure 9:**
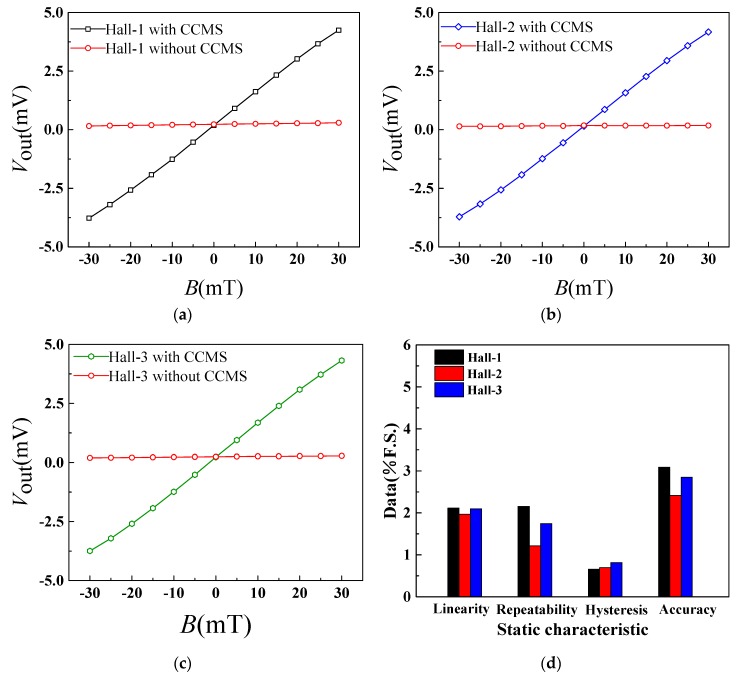
The magnetic sensitive characteristic curves of three Hall elements along the non-sensitive axis with or without CCMFS: (**a**) between *V*_out_ and *B* of Hall-1; (**b**) between *V*_out_ and *B* of Hall-2; (**c**) between *V*_out_ and *B* of Hall-3; (**d**) the static characteristic histograms Hall-1, -2 and -3 with CCMFS.

To study the magnetic field modulation performance of CCMFS, Hall elements were packaged with CCMFS. When *V*_DD_ = 5.0 V at room temperature, an external magnetic field *B* is applied to the three Hall elements of Hall-1, -2 and -3 along the non-sensitive axis (*x*-axis) from −30 mT to 30 mT with a step of 5 mT. By repeating three tests for the positive and negative strokes, *V*_out_ can be obtained by calculating the above average value of strokes. Figure 9a–c show the input-output characteristics curves of three elements with and without the CCMFS. This indicates that the proposed magnetic field sensor has not only positive but also negative magnetic sensitive characteristics along the non-sensitive direction, and *V*_out_ of Hall-1, -2 and -3 are closed to zero drift voltage without the CCMFS. The sensitivities of three Hall elements with CCMFS are 133.7 mV/T, 132.0 mV/T and 132.9 mV/T along the *x*-axis, respectively. Meanwhile, the magnetic sensitivity along the non-sensitive axis of the proposed sensor is higher compared with the sensitive-axis. Based on the above analysis, magnetic field conduction and amplification factors A_m_ of CCMFS can be calculated as 1.1. The static characteristic histograms of three Hall elements with CCMFS are shown in Figure 9d. Hall-2 has better static properties compared to the others, with an accuracy of 2.41% F.S. Experimental results indicate that it is possible for the CCMFS to convert a horizontal magnetic field component (*B*) of the Hall magnetic sensitive layer into a perpendicular component (*B*’), achieving the magnetic field measurement along the non-sensitive axis. Owing to the high magnetic permeability of CCMFS, the magnetic field component along the horizontal direction with the Hall magnetic sensitive layer can be converted completely. 

The magnetic field sensor achieves a non-sensitive direction measurement, with the decline in the linearity, repeatability and hysteresis of the magnetic field sensor. When CCMFS works under saturation conditions, the sensitivity of magnetic field sensor is gradually decreased due to the variations in magnetic conduction and the amplification ability, leading to the reduction in linearity and hysteresis. 

The sensitivity along the non-sensitive axis (*x*-axis) detected by the Hall elements packaged without CCMFS, is defined as cross-sensitivity. When exerting an external magnetic field along the *x*-axis, all of the cross-sensitivities for the three Hall elements are lower than 5%, and are far less than that of the magnetic field sensor. Due to the existence of error in the measurement process, the cross-sensitivity is a relative reference value, but not an absolute value. 

Table 1 shows the comparison of the static characteristics of Hall-1, -2 and -3 packaged with CCMFS along the sensitive axis (*z*-axis) and the non-sensitive axis (*x*-axis). From the simulation results, the magnetic flux density *B* and *B*’ at surface-A and at surface-A’ are about 162.9 mT and 172.1 mT, respectively. This indicates that the CCMFS can guide the direction of external magnetic flux and improve the value of magnetic flux density by about 10%. The sensitivity of the Hall element packaged with CCMFS along the non-sensitive axis (*x*-axis) is 1.1 times higher than that of the Hall element packaged without CCMFS along the sensitive axis (*z*-axis). This indicates that the experimental results for the sensitivities of the three Hall elements confirms the simulation results.

Table 2 shows a comparison of the performance of the magnetic concentrators (or CCMFS). In this work, the CCMFS with an L-shaped structure is characterized by a gradual reduction of the cross-section area. Due to the consistency of sensitivity in three axis directions and angle measurement in the designed 3D magnetic sensor, it is not necessary that it adopt a big gain of magnetic field. Common 3D Hall magnetic field sensors are composed of horizontal Hall elements and vertical Hall elements, resulting in additional cross-sensitivity. This effect is caused by the fabrication technology and is difficult to reduce [19]. However, by applying the CCMFSs to the 3D Hall magnetic field sensor, which is composed of the same Hall elements, makes it possible to reduce the cross-sensitivity because of the directional characteristic of magnetic flux conduction of CCMFS. In addition, 3D magnetic field sensors with the same components have better sensitivity consistency along three vector directions. 

### 4.3. Temperature Characteristic of the Magnetic Field Sensor 

**Figure 10:**
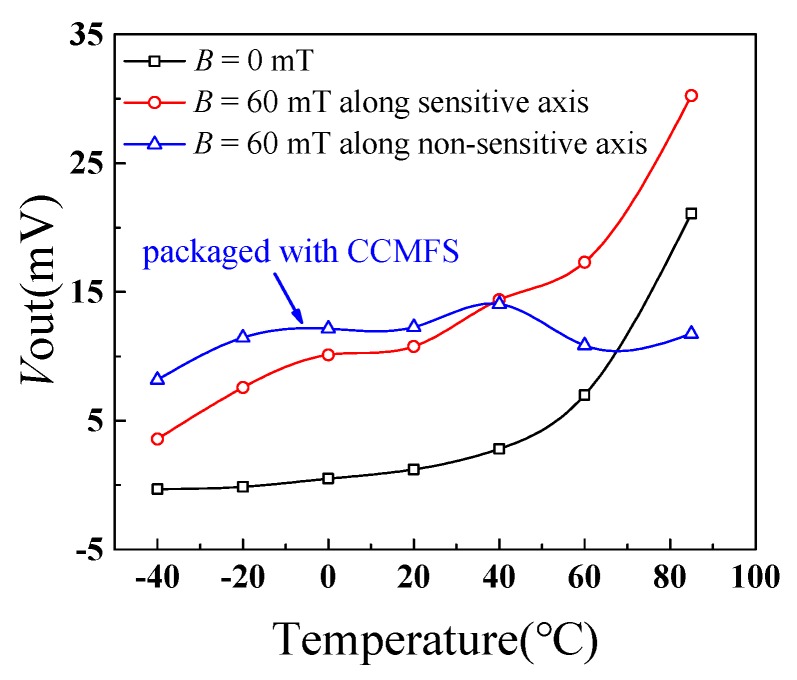
The *V*_out_ and T relationship curves of the proposed magnetic field sensor.

To study the effect of temperature on the characteristics of the magnetic field sensor, Hall-2 with CCMFS was tested by using a high-low temperature experiment chamber (GDJS-100G). Figure 10 shows the relationship curves of the proposed magnetic field sensor between *V*_out_ and temperature *T* when *V*_DD_ = 5.0 V and an increasing temperature range from −40 °C to 85 °C with a step of 20 °C. When applying the external magnetic field *B* = 0 T, the zero drift voltage values of Hall-2 packaged without CCMFS increased with increasing temperature, meaning a positive temperature coefficient, while for *B* = 60 mT along a sensitive axis (*z*-axis), the output voltage values of Hall-2 packaged without CCMFS had a positive temperature coefficient in the same way; For *B* = 60 mT along the non-sensitive axis (*x*-axis), the output voltage values of Hall-2 packaged with CCMFS increased first and then decreased. From −40 °C to 40 °C, the output voltage along the non-sensitive axis (*x*-axis) is higher than the sensitive axis (*z*-axis); and from 40 °C to 85 °C, the output voltage along the non-sensitive axis (*x*-axis) is lower than the sensitive axis (*z*-axis). At about 65 °C, the Hall-2 packaged with CCMFS loses its magnetic sensitive characteristic first, and then recovers. The change of output voltage indicates that the temperature affects the performance of the CCMFS. When the temperature increases to more than 40 °C, the magnetic field modulation performance of CCMFS declines.

## 5. Conclusions

In conclusion, a magnetic field sensor with a new concentrating-conducting magnetic flux structure (CCMFS) was designed by using a silicon-on insulator (SOI) Hall element fabricated by complementary metal oxide semiconductor (CMOS) technology as a magnetic sensitive unit. To analyze the inner magnetic field vector distribution of the SOI, a simulation model of CCMFS was built based on finite element software, where the CCMFS was processed using Ni-Fe alloys material based on a low speed wire-cut electric discharge technology. The size of the CCMFS was optimized and fixed above the Hall element, achieving a non-magnetized package on a PCB. At room temperature, the characteristics of the proposed sensor were tested by using a high precision magnetic field generating system (CH-100) and high-low temperature experiment chamber (GDJS-100G). The test results showed that when *V*_DD_ = 5.0 V, the magnetic sensitivity along the non-sensitive axis was 132 mV/T approximated to the sensitive axis in the magnetic field range from −30 mT to 30 mT. The simulation results confirmed the experimental results, indicating that it is feasible to modulate the direction of magnetic flux by using the resulting CCMFS, which is necessary to achieve the measurement of magnetic field along a non-sensitive axis. This study makes it possible to not only realize the detection of magnetic field but also to significantly improve the sensitivity of the sensor along a non-sensitive axis.

## Figures and Tables

**Table 1:** Comparison of the static characteristics of Hall-1, Hall-2 and Hall-3.

	Sensitivity	Linearity	Repeatability	Hysteresis	Accuracy
Hall-1	122.8 mV/T	0.29% F.S	1.18% F.S	0.47% F.S	1.30% F.S
Hall-1 (CCMFS)	133.7 mV/T	2.11% F.S	2.15% F.S	0.65% F.S	3.09% F.S
Hall-2	119.4 mV/T	0.14% F.S	0.25% F.S	0.11% F.S	0.31% F.S
Hall-2 (CCMFS)	132.0 mV/T	1.97% F.S	1.21% F.S	0.69% F.S	2.41% F.S
Hall-3	122.3 mV/T	0.50% F.S	1.34% F.S	0.71% F.S	1.60% F.S
Hall-3 (CCMFS)	132.9 mV/T	2.09% F.S	1.74% F.S	0.81% F.S	2.84% F.S

**Table 2:** The performance comparison of the magnetic concentrators.

Reference Parameters	Shape	Magnetic Field Gain	Direction		Applied Magnetic Field Sensor
			*B* (Before)	*B*’ (After)	
[8]	Circle shape	1.5	*B_x_* (*B_y_*)	*B_z_*	Hall elements
[6]	Slope shape	2.74	*B_z_*	*B_x_* (*B_y_*)	GMR
This work	L-shape	1.1	*B_x_* (*B_y_*)	*B_z_*	Hall element

## References

[1] Zhao X.F., Jin C.C., Deng Q., Lv M.W., Wen D.Z. (2018). Fabrication Technology and Characteristics Research of a Monolithically Integrated 2D Magnetic Field Sensor Based on Silicon Magnetic Sensitive Transistors. Sensors.

[2] Zhao X.F., Yang X.H., Li B.Z., Wen D.Z. (2016). Characteristics Research of the Three Dimensional Hall Magnetic Field Sensor Based on Packaging Technology. Sens. Transducers.

[3] Zhao X.F., Bai Y.J., Deng Q., Ai C.P., Yang X.H., Wen D.Z. (2017). Research of the Monolithic Integrated 3-D Magnetic Field Sensor Based on MEMS Technology. IEEE Sens. J..

[4] Chen J., Wurz M.C., Belski A., Rissing L. (2012). Designs and Characterizations of Soft Magnetic Flux Guides in a 3-D Magnetic Field Sensor. IEEE Trans. Magn..

[5] Schott C., Racz R., Manco A., Simonne N. (2007). CMOS Single Chip Electronic Compass with Microcontroller. IEEE J. Solid-State Circuits.

[6] Zhao J., Hu J., Tian W., Hu J., Pan M. (2015). Designs of Novel Magnetic Flux Guides for Three-Axis Magnetic Sensor. IEEE Trans. Magn..

[7] Zhao J., Tian W., Zhang Q., Pan M., Hu J., Chen D., Luo F. (2013). Designs of Slope Magnetic Flux Guides for Three-Axis Magnetic Sensor. IEEE Trans. Magn..

[8] Schott C., Huber S. (2008). Modern CMOS Hall Sensors with Integrated Magnetic Concentrators. Lect. Notes Electr. Eng..

[9] Leroy P., Coillot C., Roux A.F., Chanteur G.M. (2006). High Magnetic Field Amplification for Improving the Sensitivity of Hall Sensors. IEEE Sens. J..

[10] Wang S., He T., Zhang Y. (2014). Research on a Superconducting Magnetic Flux Concentrator for a GMI-Based Mixed Sensor. IEEE Trans. Appl. Supercond..

[11] Sun X., Jiang L., Pong P.W.T. (2013). Magnetic Flux Concentration at Micrometer Scale. Microelectron. Eng..

[12] Brugger S., Paul O. (2009). Field Concentrator Based Resonant Magnetic Sensor with Integrated Planar Coils. J. Microelectromech. Syst..

[13] Brugger S., Paul O. (2010). Magnetic Field Amplification by Slender Cuboid Shaped Magnetic Concentrators with a Single Gap. Sens. Actuators A Phys..

[14] Leitao D.C., Gameiro L., Silva A.V., Cardoso S., Freitas P.P. (2012). Field Detection in Spin Valve Sensors Using CoFeB/Ru Synthetic Antiferromagnetic Multilayers as Magnetic Flux Concentrators. IEEE Trans. Magn..

[15] Bean C P. (1962). Magnetization of Hard Superconductors. Phys. Rev..

[16] Osborn J A. (1945). Demagnetizing Factors of The General Ellipsoid. Phys. Rev..

[17] Leroy P., Coillot C., Mosser V., Roux A., Chanteur G. (2008). An ac/dc Magnetometer for Space Missions: Improvement of a Hall Sensor by the Magnetic Flux Concentration of the Magnetic Core of a Searchcoil. Sens. Actuators A Phys..

[18] Hall E.H. (1884). The Hall Effect. Science.

[19] Beran P., Stahl-Offergeld M., Peters V., Krause D., Hohe H.P. (2019). Impact of Contact Misalignment on Magnetic Cross Sensitivity of Integrated Vertical Hall Sensors. IEEE Trans. Magn..

